# Confidence-Aware Object Detection Based on MobileNetv2 for Autonomous Driving

**DOI:** 10.3390/s21072380

**Published:** 2021-03-30

**Authors:** Wei Li, Kai Liu

**Affiliations:** School of Computer Science and Technology, Xidian University, Xi’an 710071, China; wli_6@stu.xidian.edu.cn

**Keywords:** artificial neural networks, deep learning, object detection

## Abstract

Object detection is an indispensable part of autonomous driving. It is the basis of other high-level applications. For example, autonomous vehicles need to use the object detection results to navigate and avoid obstacles. In this paper, we propose a multi-scale MobileNeck module and an algorithm to improve the performance of an object detection model by outputting a series of Gaussian parameters. These Gaussian parameters can be used to predict both the locations of detected objects and the localization confidences. Based on the above two methods, a new confidence-aware Mobile Detection (MobileDet) model is proposed. The MobileNeck module and loss function are easy to conduct and integrate with Generalized-IoU (GIoU) metrics with slight changes in the code. We test the proposed model on the KITTI and VOC datasets. The mean Average Precision (mAP) is improved by 3.8 on the KITTI dataset and 2.9 on the VOC dataset with less resource consumption.

## 1. Introduction

Object detection is a basic task in computer vision. Many object detection methods have been proposed in the past few decades. Early methods mainly rely on handcrafted features and shallow machine learning algorithms, which are easy to overfit. As a result, the performance is not satisfactory. In recent years, deep artificial neural networks have been emerged and become the dominant methods in object detection problems.

Deep artificial neural networks for object detection can be roughly divided into two categories: one-stage models and two-stage models. Two-stage models have better performance, but they consume more computational resources. The one-stage models consume less resources, but their performance is not as good as that of the two-stage models. Both one-stage models and two-stage models can be used for autonomous driving and for different purposes. For two-stage models, they can be deployed on higher performing hardware. Some one-stage models can be deployed on mobile devices. In this paper, we propose Conf-MobileDet, a Confidence-aware one-stage object Detection model based on the MobileNetv2 [1] backbone for autonomous driving.

Object detection for autonomous driving involves accurate detection ability in traffic scenes. Objects to be detected mainly consist of vehicles, pedestrians, and cyclists. In addition, objects in traffic scenes are often very dense and of various sizes. Many objects are occluded by others. In some cases, objects used for training are not well labeled. These phenomena inevitably make the model learn ambiguous features and decrease the localization confidence. Traditional object detection models output definite detected object positions, the objectness score, and their classes. These outputs cannot quantify the positioning confidence.

To address these problems, we propose a MobileNeck module and use a cross-entropy loss function for learning bounding box regression and localization confidence based on Generalized-IoU (GIoU) [2] loss. Specifically, to obtain the confidence of bounding box prediction, we model the bounding box prediction and bounding box labels as Gaussian distributions and Dirac delta functions, respectively. The new loss can be defined by a KL divergence with respect to the distribution of the bounding box prediction and the bounding box labels. The loss function is equal to a cross-entropy loss function. We use this cross-entropy loss function to model the confidence of the bounding box prediction. Moreover, we combine the proposed loss function with the truncated MobileNetv2 backbone and MobileNeck module to test the proposed methods.

The contributions of our work are as follows:

1. We designed a multi-scale MobileNeck module that was used to further extract higher dimensional features and enlarge the effective receptive field [3].

2. We used a cross-entropy loss function for learning bounding box regression and localization confidence based on the GIoU loss function.

3. We applied the proposed methods to the MobileNetv2 backbone. Experiments were performed on the KITTI and Pascal VOC datasets, which showed that the proposed model achieved better performance compared to the original methods.

## 2. Related Work

Many object detection models have been proposed for autonomous driving. Traditional methods rely on hand-crafted features and shallow machine learning algorithms, which easily overfit. As a result, the performance is not promising. Convolutional neural networks (CNNs) extract object features autonomously and simultaneously use a linear classifier to predict the object locations and classes. The result outperforms traditional methods by a large margin.

CNNs for object detection can be approximately divided into two classes: one-stage models and two-stage models. Two-stage models generate a sparse object candidate set in the first stage and predict objects on the sparse set in the second stage. The representative works of two-stage models include R-CNN [4] and their descendants, such as Fast R-CNN [5], Faster R-CNN [6], Mask R-CNN [7], and Cascade R-CNN [8].

Different from two-stage models, one-stage models directly predict object locations and classes based on predefined dense anchor boxes and treat the object detection task as a regression problem. The representative works of one-stage models include MobileNet [1,9,10], SqueezeDet [11], Single Shot Multibox Detector (SSD) [12], YOLO [3,13,14,15], EfficientDet [16], GhostNet [17], and their descendants.

Our purpose is to design an object detection model that can be deployed on mobile devices. MobileNet and its descendants are designed for these tasks. MobileNetv2 is a recent representative object detection model, so our work is based on MobileNetv2.

In addition to carefully designed models for mobile devices, some researchers are working toward the design of various loss functions for their specific use. One-stage object detection models predict thousands of bounding boxes and corresponding classes in which many of them belong to the negative samples. The contribution from positive samples to the loss function is easily overwhelmed by that of negative samples. SSD resolves this problem by heuristic hard negative mining. RetinaNet [18] resolves this problem by modifying the traditional cross-entropy loss function. They propose focal loss to tackle the imbalance of positive samples and negative samples. In RetinaNet, focal loss can filter out easy examples, leaving hard samples to train a discriminative network. As a result, the negative effects introduced by the imbalance of positive samples and negative samples are resolved. In this work, we use focal loss for classification. The proposed methods can work well together with focal loss.

The traditional loss function for bounding box regression is the smooth $L_{1}$ loss. It independently regresses the central point coordinates, width, and height with respect to the predefined anchor boxes. In addition, the actual evaluation target is the Intersection over Union (IoU). They are not absolutely equivalent. To compensate for the gap between the smooth $L_{1}$ loss and IoU, Zhou et al. proposed IoU loss [19], which regresses the offset between the location of the object’s center and the center of an anchor box and then uses the width and height of the anchor box to predict the relative scale of the predicted object boxes. Apart from IoU loss, Generalized-IoU (GIoU) loss [2], Distance-IoU (DIoU) loss [20], and Complete-IoU (CIoU) loss [20] were proposed, which can be seen as extending IoU loss. In this work, we used the proposed methods to improve the performance of GIoU loss. It is easy to conduct, and the modification can work well together with the original GIoU loss. Since the objectiveness score is used to reflect the possibility of whether an object exists, it cannot reflect the quality of the predicted bounding boxes well. We need better means to mend the deficiency. This problem is resolved by bounding box confidence-based Non-Maximum Suppression (NMS).

Our work is related most to [21,22], which output Gaussian parameters for bounding box regression. However, there were three differences.

1. During the design of the proposed model, we considered that the model should be applicable to mobile devices. Based on this prerequisite, we designed the light-weight multiscale MobileNeck network. It was used to further extract higher dimensional features and enlarge the effective receptive field.

2. The proposed model contains fewer multiply-add operations compared to [21,22].

3. We illustrate the generated unconfidence degree in the object detection results. In addition, explanations are added to describe the rationality of proposed methods.

## 3. Materials and Methods

In this section, first, we describe the Conf-MobileDet architecture. Next, we propose the bounding box parameterization and the cross-entropy loss-based bounding box confidence prediction. Finally, we introduce a confidence-based NMS approach.

### 3.1. Model Architecture

**Figure 1:**
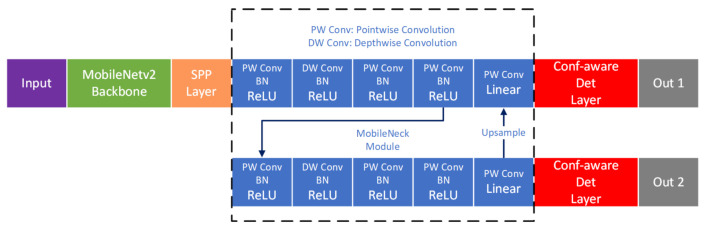
The proposed model. SPP, Spatial Pyramid Pooling; Conf-aware, Confidence-aware; Det, detection.

The proposed model is composed of four parts: MobileNetv2 backbone, Spatial Pyramid Pooling (SPP) layer, MobileNeck layers, and object detection layers. The model is shown in Figure 1.

We used the truncated MobileNetv2 model as our backbone network. The basic MobileNetv2 backbone was built on a depthwise separable convolution layers and pointwise convolution layers, which made the backbone contain fewer multiply-add operations during each forward propagation. The layer parameters are in Table 1. It was pretrained on ImageNet and used to extract high-dimensional features for each input image.

The SPP layer takes the output of the truncated MobileNetv2 model. It pools feature maps of various sizes and outputs feature maps that have the same sizes in spatial dimensions. Based on SPP, the proposed model can take images of different sizes during both the training and evaluation stages.

**Figure 2:**
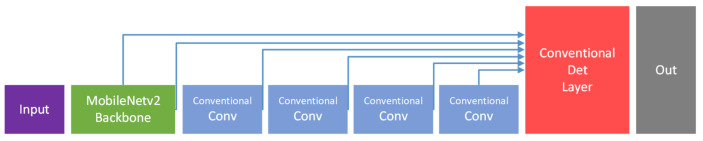
MobileNetv2 with the SSDdetector.

Since MobileNeck is built with depthwise separable convolutions and pointwise convolutions and is located between the backbone and the detection head, we call it MobileNeck. Compared to SSDNeck layers built with conventional convolution layers (Figure 2), MobileNeck imposes a lower computational burden. We used upsampling and skip connections to strengthen the semantic information in the first detection branch.

The detection layer of the proposed model is confidence-aware. It is responsible for predicting the confidence of each output with bounding box coordinates, objectness scores, and classification scores. As a result, the dimension of each output is (c+8+1)∗n compared to conventional (c+4+1)∗n, where *c* is the number of classes in the dataset and *n* is the number of anchors at each pixel on the output feature maps. It is 4∗n longer than conventional outputs. These additional outputs are used to predict the confidence scores of each bounding box.

### 3.2. Model Parameters

**Figure 3:**
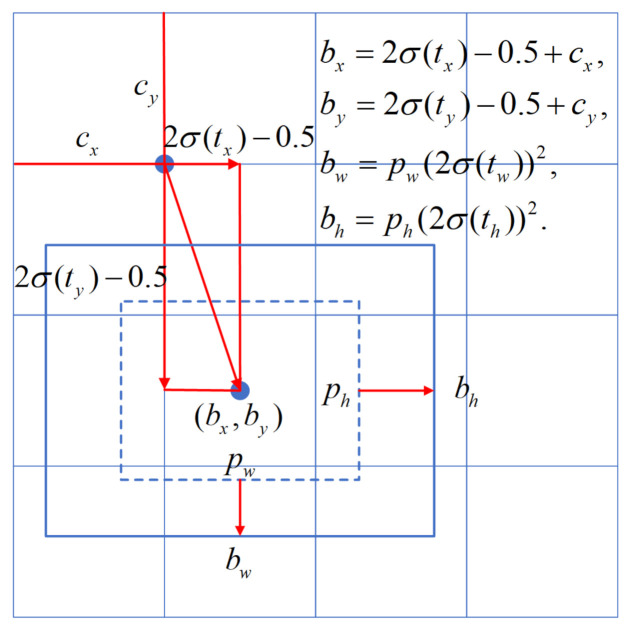
Bounding box regression in the proposed model.

Figure 1 shows the proposed model. It regresses the two-dimensional top-left point $\left(x_{c},y_{c}\right)$ and width, height (w,h) of a bounding box independently.

(1) $$\begin{array}{c} \begin{array}{cc} b_{x} & =2\sigma \left(t_{x}\right)-0.5+c_{x},\\ b_{y} & =2\sigma \left(t_{y}\right)-0.5+c_{y},\\ b_{w} & =p_{w}{\left(2\sigma \left(t_{w}\right)\right)}^{2},\\ b_{h} & =p_{h}{\left(2\sigma \left(t_{h}\right)\right)}^{2}. \end{array} \end{array}$$

where $b_{*}$ denotes the predicted bounding boxes, $t_{*}$ is the output of the proposed network, $c_{*}$ is the offset of a cell from the top-left corner of the feature map, and $p_{*}$ is the width or height of the predefined anchor. Different from [21], in which the offsets of $c_{x}$ are confined within the boundaries of their grids and may lead to the grid sensitivity phenomenon [3], we used the metrics described in Equation 1 to prevent the problem [3]. In the equation, the output of the network $t_{*}$ is multiplied by a factor that is greater than one. This allows the offset of predicted bounding boxes to be larger than its original grid. Figure 3 shows the bounding box regression process.

The objective is to estimate the confidence score of the predicted location. Similar to classification score prediction, the network needs to predict a probability distribution along with a definite object location. Because the predicted central point coordinates, box width, and height are independent, we can use a single-variate Gaussian model for simplicity:

(2) $$\begin{array}{c} \begin{array}{c} f_{\Theta }\left(x\right)=\frac{1}{\sqrt{2\pi }\sigma }e^{\frac{{\left(x-\mu \right)}^{2}}{2\sigma^{2}}}, \end{array} \end{array}$$

where Θ is the trainable network parameter set, μ is the predicted bounding box location, and σ stands for the degree of unconfidence for the predicted bounding box location. The bounding box confidence can be formulated as conf=1−σ,(0<σ<1). For conf→1, the network becomes extremely confident about the predicted bounding box location. μ and σ are the outputs of the last layer of the network. They are produced by a convolutional layer. Figure 1 shows the proposed architecture.

**Figure 4:**
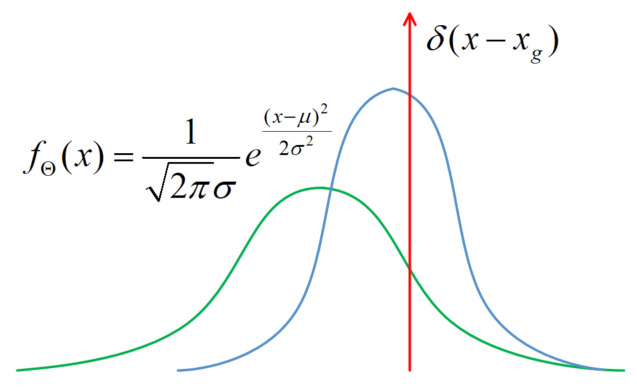
Gaussian model and its parameters used for bounding box regression.

The bounding box labels can also be formulated as a Gaussian distribution with σ→0, which is a delta function:

(3) $$\begin{array}{c} \begin{array}{c} \delta \left(x-x_{g}\right)=\left\{\begin{array}{cc} 1 & x=x_{g},\\ 0 & \mathrm{otherwise}, \end{array}\right. \end{array} \end{array}$$

where $x_{g}$ denotes the bounding box labels. The relationship between Equations (2) and (3) and the model parameters is shown in Figure 4.

### 3.3. Bounding Box Regression with Cross-Entropy Loss

We used the cross-entropy loss function to model the localization confidences of predicted bounding boxes over a single sample:

(4) $$\begin{array}{c} \begin{array}{cc} L_{reg} & =H(p_{D}\left(x\right),p_{\Theta }\left(x\right))\\ \; & =-\int p_{D}\left(x\right)p_{\Theta }\left(x\right)\mathrm{dx}\\ \; & =-\int \delta \left(x-x_{g}\right)f_{\Theta }\left(x\right)\mathrm{dx}\\ \; & =\frac{{\left(x_{e}-x_{g}\right)}^{2}}{2\sigma^{2}}+\frac{\log(\sigma^{2})}{2}+\frac{\log(2\pi )}{2} \end{array} \end{array}$$

where H(∗,∗) is cross-entropy loss function; $p_{D}\left(x\right)$ is equal to $\delta (x-x_{g})$ and denotes the ground truth of the object’s coordinates; $p_{\Theta }\left(x\right)$ is equal to $f_{\Theta }\left(x\right)$, and it is a Gaussian distribution based on the output of the Gaussian parameters. By deriving the formula, we get $L_{reg}=\frac{{\left(x_{e}-x_{g}\right)}^{2}}{2\sigma^{2}}+\frac{\log(\sigma^{2})}{2}+\frac{\log(2\pi )}{2}$. Because $\frac{\log(2\pi )}{2}$ is a constant, the loss function can be rewritten in a simple form:

(5) $$\begin{array}{c} \begin{array}{c} L_{reg}=\frac{{\left(x_{e}-x_{g}\right)}^{2}}{2\sigma^{2}}+\frac{\log(\sigma^{2})}{2} \end{array} \end{array}$$

If $\left|(x_{e}-x_{g})\right|$ is large, the network is unconfident about the prediction. In this condition, we expect the network outputs to be large σ, so that the loss will become small (Figure 4). For $\sigma^{2}=1$, $\frac{\log(\sigma^{2})}{2}$ is equal to 0. The cross-entropy loss degenerates to the normal Euclidean bounding box regression loss:

(6) $$\begin{array}{c} \begin{array}{c} L_{reg}={\left(x_{e}-x_{g}\right)}^{2} \end{array} \end{array}$$

As a result, the proposed model is an extension of the normal Euclidean bounding box regression model. For the batch training method, assuming there are M samples in a batch, the loss function for a batch of data is:

(7) $$\begin{array}{c} \begin{array}{c} \hat{\Theta }=\frac{1}{M}\arg\min_{\Theta }\sum H(p_{D}\left(x\right),p_{\Theta }\left(x\right)). \end{array} \end{array}$$

The goal of the localization loss function is to estimate the trainable weight set $\hat{\Theta }$ that minimizes the cross-entropy loss $L_{reg}$ over M samples.

### 3.4. NMS Based on Bounding Box Confidence

Conventional NMS eliminates overlapping bounding boxes by comparing the objectness score in a local region. It reserves only one bounding box that has the maximum objectness score. There is a gap between the objectness score and bounding box positioning because the objectness score mainly reflects the classification quality, and it is not equivalent to how precise the position of the predicted bounding boxes is. We used a confidence score to remedy it. We revised the objectness score by integrating confidence scores by the following equation, which considers both the objectness score and confidence scores:

(8) $$\begin{array}{c} \begin{array}{c} Object\_Score=\sqrt{Object\_Score*Conf\_Score}. \end{array} \end{array}$$

where 0≤Object_Score≤1, 0≤Conf_Score≤1. The radical sign in the equation is used to prevent the multiply results from becoming too small. We found using the radical sign in the equation led to better performance.

## 4. Results

The proposed model was evaluated on the KITTI [23] and Pascal VOC [24] datasets. We analyzed the performance by mAP (mean Average Precision) and computational resource consumption (Gflops, inference time). At the end of the experiment, the object detection result was used to analyze how the predicted degree of unconfidence affected the loss function to prove the rationality of the proposed methods. We carried out experiments with a single NVIDIA 1080 Ti GPU, 8 GB of main memory, a Core i7 processor (3.2 GHz), and an Ubuntu 18.04 operating system.

### 4.1. Experiments on the KITTI Dataset

KITTI is an object detection dataset for autonomous driving. It contains 7381 training images with ground truth labels. There are three object categories in KITTI: vehicle, person, and cyclist. The objects to be detected are of various sizes and contain many small samples, which make it a challenging task. KITTI allows users to use it under the terms and conditions of the Creative Commons Attribution (CC BY) license. Users can copy, redistribute the material in any medium or format, and remix, transform, and build upon the material.

The training was composed of two stages. In the first stage, the learning rate increased from $10^{-6}$ slowly after it reached 0.001. In this stage, the truncated MobileNetv2 network was frozen, and only the MobileNeck layers were updated. In the second stage, the whole network was updated. The Stochastic Gradient Descent (SGD) optimizer was used. The learning rate was divided by 10 after the 80,000th and 100,000th iterations. The training was stopped at the 120,000th iteration. We scaled the input image size into various sizes such as 320×320 and 352×352 in the training stage. It took about 20 h to converge. In the evaluation stage, the input image size was scaled to 520×520 to achieve better performance. Because the labels of the test set were not publicly available, similar to SqueezeDet, we randomly split the 7381 training images in half into a training set and a validation set. All results were on the validation set. The batch size was eight. The backbone layer weights used for initializing the model were pretrained on ImageNet. Data augmentation techniques such as random cropping and flipping were adopted to avoid overfitting.

The performance of the models was evaluated in terms of mAP and Gflops (Giga floating-point operations per second). We re-evaluated MobileNev2 with the SSD detection head, MobileNetv2 with MobileNeck, and its detection confidence-aware version for a fair comparison. In addition, other studies performed training using the default settings in the official code of each algorithm. To test the effect of MobileNeck and the confidence-aware loss function, we conducted a control experiment. First, we tested MoileNetv2 with the SSD detection head model. Second, we tested MobileNetv2 with MobileNeck and conventional loss. In this experiment, the loss was the same as that used in the first experiment. Third, we modified the loss function in the second experiment. We used the confidence-aware version in this experiment. The performance for each configuration is shown in Table 2.

In the table, both the MobileNeck structure and confidence-aware loss have positive effects on the MobileNetv2-based object detection model. Compared to MobileNetv2 with the SSD detector, the proposed model outperformed it by 3.8 percentage points in terms of mAP with fewer Gflops.

We compare the proposed model with several classical lightweight deep artificial neural networks (SSD, YOLOv3, Gaussian YOLOv3, SqueezeDet, SqueezeDet+) in Table 3.

**Figure 5:**
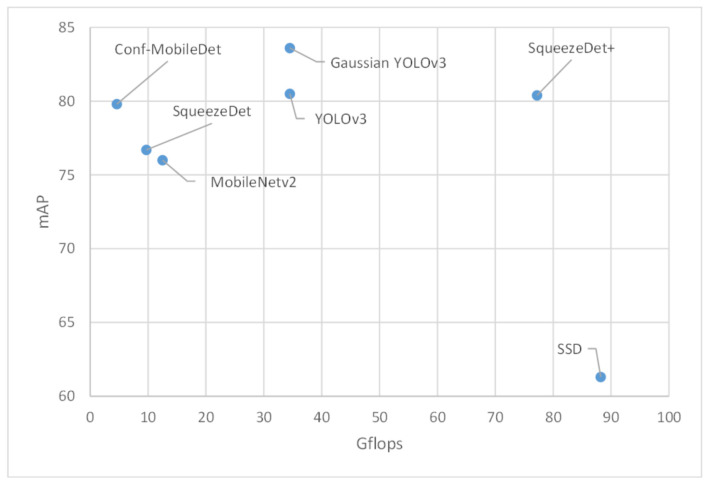
Scatter plot comparing Gflops and detection accuracy of different methods. mAP, mean Average Precision.

In the table, the proposed model has better performance than most other models in the table. Compared to SqueezeDet, which is the representative object detector designed for mobile devices, Conf-MobileDet outperformed it by 3.1 percentage in terms of mAP evaluation metrics and consumed fewer Gflops. Although SqueezeDet+ achieved better performance than Conf-MobileDet by 0.6 percentage points, its Gflops were 16 times those of Conf-MobileDet. For clarity, a scatter plot is drawn to compare different methods, whose horizontal and vertical ordinates are the Gflops and detection accuracy, respectively (Figure 5). As a result, Conf-MobileDet is more suitable for mobile devices that have only limited computational resources.

**Figure 6:**
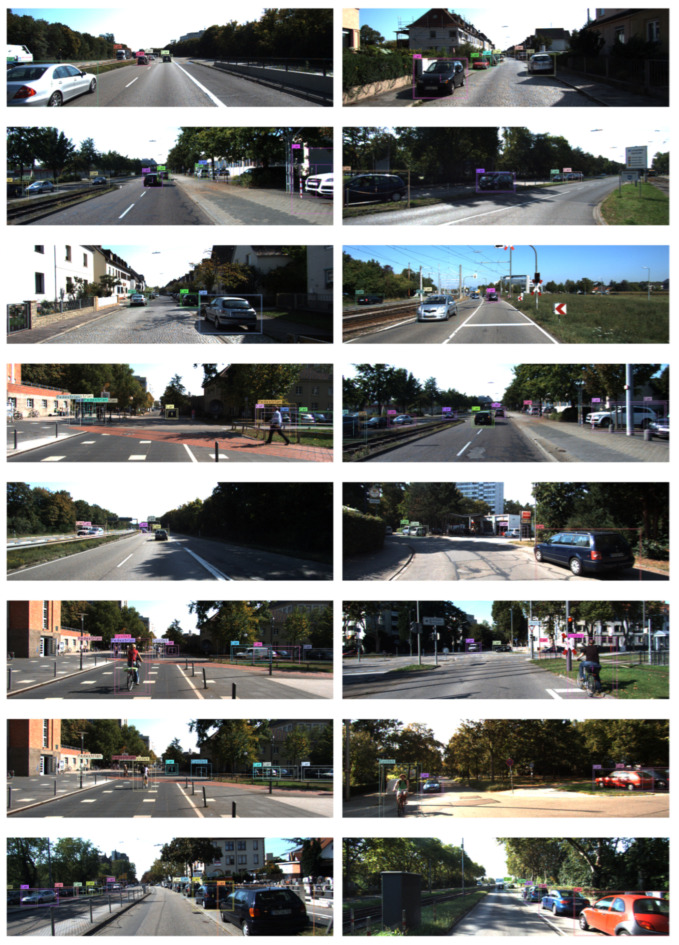
Object detection result on the KITTI dataset. It is better to zoom in the figures before viewing.

The detection result is shown in Figure 6. The predicted bounding boxes are represented by dashed rectangles. The predicted degree of unconfidence for the box size is represented by solid rectangles around the dashed rectangles, and the degree of unconfidence for box center coordinates is visualized by crossed lines inside the dashed rectangles. In order to facilitate the observation of the predicted degree of unconfidence, they are mapped to the exponential space:

(9) $$\begin{array}{c} \begin{array}{cc} {\hat{uc}}_{x} & =min(exp\left(uc_{x}*b\right),w/2.0),\\ {\hat{uc}}_{y} & =min(exp\left(uc_{y}*b\right),h/2.0),\\ {\hat{uc}}_{w} & =min(exp\left(uc_{w}*b\right),w/2.0),\\ {\hat{uc}}_{h} & =min(exp\left(uc_{h}*b\right),h/2.0), \end{array} \end{array}$$

where $uc_{*}$ is the degree of unconfidence. *b* is a weighting factor, and we set b=15 in this experiment. min(∗,∗) is used to prevent the boundary of the solid rectangle and cross lines from deviating too much from their common center point. It can be seen that for blurred objects or objects that are occluded by others, the offsets of their corresponding solid rectangles and cross lines from the their common center point are larger than the objects that are easy to distinguish. Therefore, the loss in Equation (7) for difficult samples will be larger than that of easy samples accordingly. This will cause the network to converge in a direction that minimizes the losses provided by these difficult samples. It is better to zoom in the figures before viewing.

**Figure 7:**
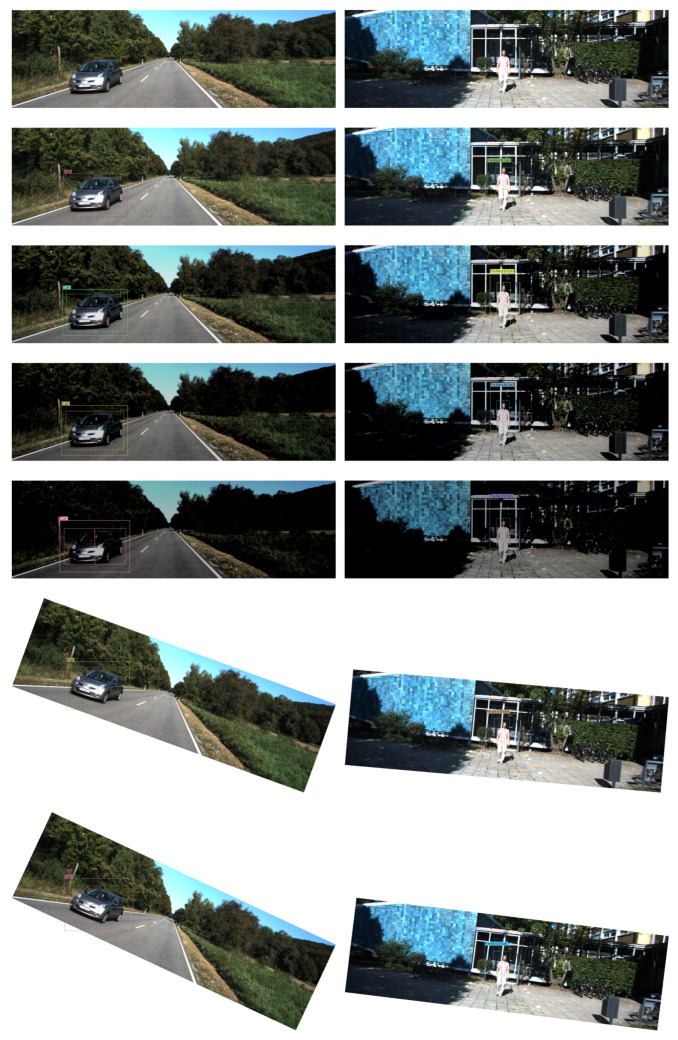

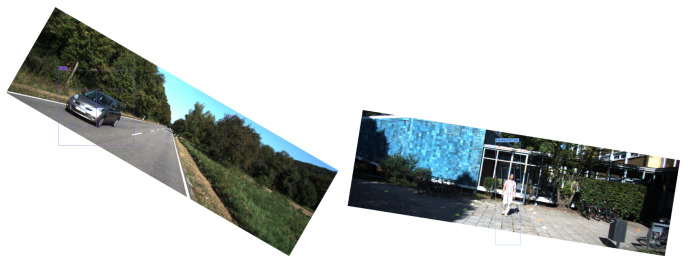
Object detection result on the KITTI dataset under different circumstances. It is better to zoom in the figures before viewing.

We extended the experiment under different circumstances. We introduced changes to the images (first line in Figure 7) such as illumination, rotation, translation, etc. We found that changes in illumination and rotation had a great impact on the original results (The second line in Figure 7). We gradually reduced the illumination (the third line to the fifth line in Figure 7) and increased the rotation angle (the sixth line to the eighth line in Figure 7) to the original images. We found that the degree of unconfidence increased gradually. This experiment indicated that adequate illumination and correct angles were important for the safety of autonomous driving as it output a low degree of unconfidence.

### 4.2. Experiment on the Pascal VOC Dataset

We also tested the proposed methods on the Pascal VOC dataset. Because inference time is essential for autonomous driving, in this experiment, we compared MobileNetv2 and Conf-MobileDet by mAP and inference time. The Pascal VOC dataset contains three computer vision tasks: classification, detection and segmentation. The object detection task has 20 different classes to be detected, such as cats, houses, and persons. We used the VOC 07+12 train and valsets for training and the VOC 07 test set for evaluation. In this experiment, the backbone was pretrained on the ImageNet dataset and fine-tuned on the Pascal VOC dataset.

The training was composed of two stages. In the first stage, the learning rate slowly increased from $10^{-6}$ after it reached 0.0001. In this stage, the truncated MobileNetv2 network was frozen, and only the MobileNeck layers were updated. In the second stage, the whole network was updated. The SGD optimizer was used. The learning rate was divided by 10 after 80,000 and 100,000 iterations. The training was stopped at 120,000 iterations. We scaled the input image size into various sizes, such as 320×320 and 352×352 in the training stage. It took about 12 h to converge. In the evaluation stage, the input image size was scaled to 320×320 for fast reference speed. We list the mAP and inference time of MobileNetv2 model and the proposed model in Table 4.

In the table, both the MobileNeck structure and confidence-aware loss have positive effects on the MobileNetv2-based object detection model. Compared to MobileNetv2, Conf-MobileDet outperformed it by 2.9 percentage points in terms of mAP with less inference time.

**Figure 8:**
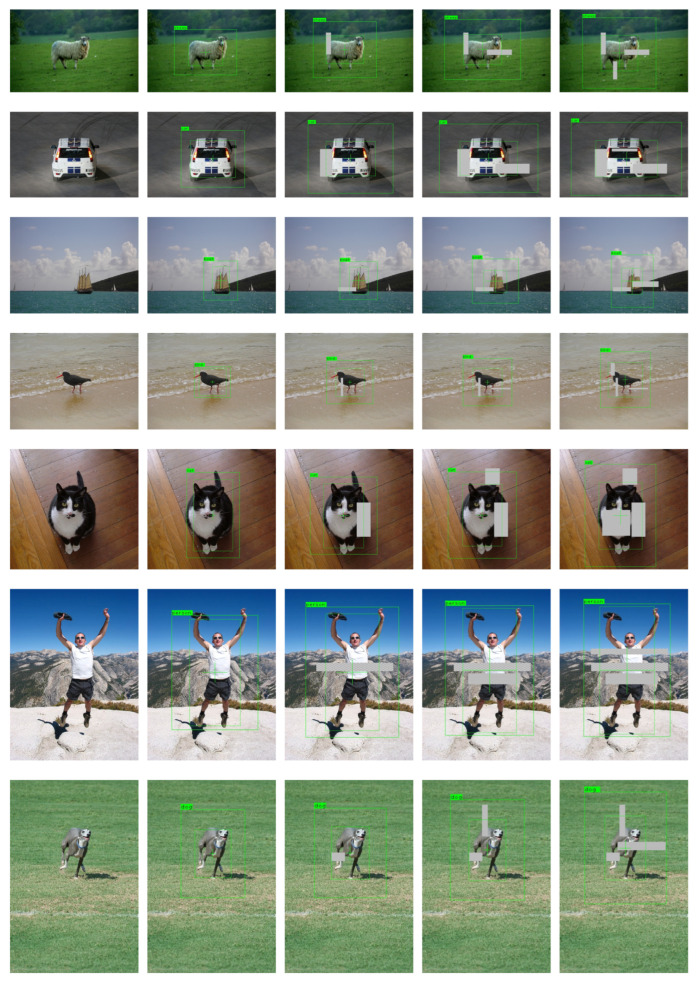
Object detection result on the Pascal VOC dataset. It is better to zoom in the figures before viewing.

The detection result on VOC dataset is shown in Figure 8. The predicted bounding boxes are represented by dashed rectangles. The predicted degree of unconfidence for box size is represented by solid rectangles around the dashed rectangles, and the degree of unconfidence for box center coordinates is visualized by crossed lines inside the dashed rectangles. In order to facilitate the observation of the predicted degree of unconfidence, they are mapped to the exponential space before drawing. We set b=12 in this experiment (Equation (9)). It can be seen that as more occlusions are added, the predicted degree of unconfidence will be higher. This is the way the predicted degree of unconfidence impacts the confidence-aware loss function. The higher the degree of unconfidence, the larger the loss is. The computational resources will tilt to these “hard” samples. It is better to zoom in the figures before viewing.

## 5. Discussion

In this paper, an object detection architecture based on MobileNeck and confidence-aware loss was proposed. We evaluated MobileNeck and confidence-aware loss on the KITTI and Pascal VOC datasets. Experimental results showed that it had better performance compared to previous mobile device-oriented object detection architectures such as MobileNetv2 and SqueezeDet. In addition, we analyzed how the degree of unconfidence impacted the training of the model. As a result, the issues mentioned in the Introduction Section were fixed.

**Figure 9:**
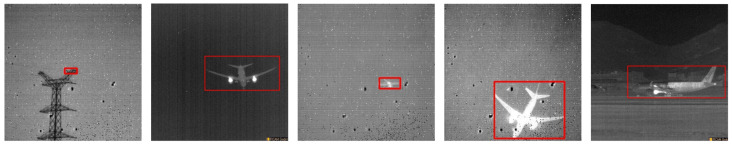
Infrared object detection result.

In the future, our challenge is to apply the proposed methods to other object detection tasks, such as infrared object detection (Figure 9). Infrared object detection are often applied to early warning systems. The objects to be detected in this task often lack rich texture features, and the background is complex. The left figure of Figure 9 is a failure example: it misjudges the background as the object. We hope the proposed method can predict the degree of unconfidence so that we can trust the result with a low degree of unconfidence and pay more attention to improving the detection result with a high degree of unconfidence, which in turn would reduce the false alarm rate of early warning systems.

## Figures and Tables

**Table 1:** Parameters of truncated MobileNetv2 used as the backbone of the proposed model.

Input	Operator	t	c	n	s
$224^{2}\times 3$	Conv2d	-	32	1	2
$112^{2}\times 32$	Bottleneck	1	16	1	1
$112^{2}\times 16$	Bottleneck	6	24	2	2
$56^{2}\times 24$	Bottleneck	6	32	3	2
$28^{2}\times 32$	Bottleneck	6	64	4	2
$28^{2}\times 64$	Bottleneck	6	96	3	1
$7^{2}\times 160$	Bottleneck	6	160	3	2

**Table 2:** Control experiment on the KITTI dataset.

Method	Car	Pedestrian	Cyclist	mAP	Gflops
MobileNetv2 + SSD [1]	89.3	65.7	73.0	76.0	12.5
MobileNetv2 + MobileNeck (ours)	90.2	68.1	78.2	78.8	4.6
Conf-MobileDet (ours)	90.4	69.5	79.4	79.8	4.6

**Table 3:** Evaluation results on the KITTI dataset.

Method	Car	Pedestrian	Cyclist	mAP	Gflops
SSD [12]	85.1	48.1	50.7	61.3	88.2
YOLOv3 [15]	79.5	83.1	79.0	80.5	34.5
Gaussian YOLOv3 [21]	87.3	79.9	83.6	83.6	34.5
SqueezeDet [11]	82.9	70.4	76.8	76.7	9.7
SqueezeDet+ [11]	85.5	73.7	82.0	80.4	77.2
Conf-MobileDet (ours)	90.4	69.5	79.4	79.8	4.6

**Table 4:** Control experiment on the Pascal VOC dataset.

Method	mAP	Inference Time (ms)
MobileNetv2 + SSD [1]	68.4	79.4
MobileNetv2 + MobileNeck (ours)	70.1	58.8
Conf-MobileDet (ours)	71.3	58.8

## Data Availability

The data presented in this study are openly available in KITTI, Pascal VOC at 10.1109/CVPR.2012.6248074, 10.1007/s11263-009-0275-4, reference number [23,24].

## References

[1] Sandler M., Howard A., Zhu M., Zhmoginov A., Chen L.C. MobileNetV2: Inverted Residuals and Linear Bottlenecks. Proceedings of the IEEE Conference on Computer Vision and Pattern Recognition.

[2] Rezatofighi H., Tsoi N., Gwak J., Sadeghian A., Reid I., Savarese S. Generalized Intersection over Union: A Metric and A Loss for Bounding Box Regression. Proceedings of the IEEE/CVF Conference on Computer Vision and Pattern Recognition.

[3] Bochkovskiy A., Wang C.Y., Liao H.Y.M. (2020). YOLOv4: Optimal Speed and Accuracy of Object Detection. arXiv.

[4] Girshick R., Donahue J., Darrell T., Malik J. Rich feature hierarchies for accurate object detection and semantic segmentation. Proceedings of the IEEE Conference on Computer Vision and Pattern Recognition.

[5] Girshick R. Fast R-CNN. Proceedings of the IEEE International Conference on Computer Vision.

[6] Ren S., He K., Girshick R., Sun J. (2016). Faster R-CNN: Towards Real-Time Object Detection with Region Proposal Networks. arXiv.

[7] He K., Gkioxari G., Dollár P., Girshick R. Mask R-CNN. Proceedings of the IEEE International Conference on Computer Vision.

[8] Cai Z., Vasconcelos N. Cascade R-CNN: Delving into High Quality Object Detection. Proceedings of the IEEE Conference on Computer Vision and Pattern Recognition.

[9] Howard A.G., Zhu M., Chen B., Kalenichenko D., Wang W., Weyand T., Adam H. (2017). MobileNets: Efficient Convolutional Neural Networks for Mobile Vision Applications. arXiv.

[10] Howard A., Sandler M., Chu G., Chen L.C., Chen B., Tan M., Adam H. Searching for MobileNetV3. Proceedings of the IEEE/CVF International Conference on Computer Vision.

[11] Wu B., Iandola F., Jin P.H., Keutzer K. SqueezeDet: Unified, Small, Low Power Fully Convolutional Neural Networks for Real-Time Object Detection for Autonomous Driving. Proceedings of the IEEE Conference on Computer Vision and Pattern Recognition Workshops.

[12] Liu W., Anguelov D., Erhan D., Szegedy C., Reed S., Fu C.Y., Berg A.C. (2016). SSD: Single Shot MultiBox Detector. European Conference on Computer Vision.

[13] Redmon J., Divvala S., Girshick R., Farhadi A. You Only Look Once: Unified, Real-Time Object Detection. Proceedings of the IEEE Conference on Computer Vision and Pattern Recognition.

[14] Redmon J., Farhadi A. YOLO9000: Better, Faster, Stronger. Proceedings of the IEEE Conference on Computer Vision and Pattern Recognition.

[15] Redmon J., Farhadi A. (2018). YOLOv3: An Incremental Improvement. arXiv.

[16] Tan M., Pang R., Le Q.V. EfficientDet: Scalable and Efficient Object Detection. Proceedings of the IEEE/CVF Conference on Computer Vision and Pattern Recognition.

[17] Han K., Wang Y., Tian Q., Guo J., Xu C., Xu C. GhostNet: More Features from Cheap Operations. Proceedings of the IEEE/CVF Conference on Computer Vision and Pattern Recognition.

[18] Lin T.Y., Goyal P., Girshick R., He K., Dollár P. Focal Loss for Dense Object Detection. Proceedings of the IEEE International Conference on Computer Vision.

[19] Zhou D., Fang J., Song X., Guan C., Yin J., Dai Y., Yang R. IoU Loss for 2D/3D Object Detection. Proceedings of the 2019 International Conference on 3D Vision (3DV).

[20] Zheng Z., Wang P., Liu W., Li J., Ye R., Ren D. Distance-IoU Loss: Faster and Better Learning for Bounding Box Regression. Proceedings of the AAAI Conference on Artificial Intelligence.

[21] Choi J., Chun D., Kim H., Lee H.J. Gaussian YOLOv3: An Accurate and Fast Object Detector Using Localization Uncertainty for Autonomous Driving. Proceedings of the IEEE/CVF International Conference on Computer Vision.

[22] He Y., Zhu C., Wang J., Savvides M., Zhang X. Bounding Box Regression with Uncertainty for Accurate Object Detection. Proceedings of the IEEE/CVF Conference on Computer Vision and Pattern Recognition.

[23] Geiger A., Lenz P., Urtasun R. Are we ready for autonomous driving? the kitti vision benchmark suite. Proceedings of the 2012 IEEE Conference on Computer Vision and Pattern Recognition.

[24] Everingham M., Van Gool L., Williams C.K., Winn J., Zisserman A. (2006). The PASCAL Visual Object Classes Challenge 2007 (VOC2007) Development Kit. http://host.robots.ox.ac.uk/pascal/VOC/.

